# Neuroprotective Efficacy of a Nanomicellar Complex of Carnosine and Lipoic Acid in a Rat Model of Rotenone-Induced Parkinson’s Disease

**DOI:** 10.3390/antiox12061215

**Published:** 2023-06-04

**Authors:** Olga Kulikova, Dmitry Troshev, Daniil Berezhnoy, Sergey Stvolinsky, Yulia Timoshina, Denis Abaimov, Olga Muzychuk, Alexander Latanov, Tatiana Fedorova

**Affiliations:** 1 Laboratory of Translational and Experimental Neurochemistry, Research Center of Neurology, 125367 Moscow, Russia; berezhnoy.daniil@gmail.com (D.B.); slstvolinsky@mail.ru (S.S.); july.timoschina@yandex.ru (Y.T.); abaidenis@yandex.ru (D.A.); muzychuk@neurology.ru (O.M.); tnf51@bk.ru (T.F.); 2 Laboratory of Neural and Neuroendocrine Regulations, Koltzov Institute of Developmental Biology, Russian Academy of Sciences, 119334 Moscow, Russia; dmitry.vad.troshev@gmail.com; 3 Department of Neurobiology, Lomonosov Moscow State University, 119991 Moscow, Russia; 4 Research Institute of Functional Brain Development and Peak Performance, Peoples’ Friendship University of Russia, 117198 Moscow, Russia

**Keywords:** neuroprotection, Parkinson’s disease, rat, antioxidants, carnosine, lipoic acid, rotenone

## Abstract

Oxidative stress, accompanied by mitochondrial dysfunction, is a key mechanism involved in the pathogenesis of Parkinson’s disease (PD). Both carnosine and lipoic acid are potent antioxidants, the applicability of which in therapy is hindered by their limited bioavailability. This study aimed to evaluate the neuroprotective properties of a nanomicellar complex of carnosine and lipoic acid (CLA) in a rotenone-induced rat model of PD. Parkinsonism was induced via the administration of 2 mg/kg rotenone over the course of 18 days. Two doses of intraperitoneal CLA (25 mg/kg and 50 mg/kg) were administered alongside rotenone to assess its neuroprotective effect. At 25 mg/kg CLA decreased muscle rigidity and partially restored locomotor activity in animals that received rotenone. Furthermore, it caused an overall increase in brain tissue antioxidant activity, accompanied by a 19% increase in neuron density in the substantia nigra and increased dopamine levels in the striatum relative to animals that only received rotenone. Based on the acquired results, it may be concluded that CLA have neuroprotective properties and could potentially be beneficial in PD treatment when used in conjunction with the base therapy.

## 1. Introduction

The second most frequent neurodegenerative pathology after Alzheimer’s disease, Parkinson’s disease (PD), is characterized by selective, irreversible, and progressive loss of dopaminergic neurons [1]. Clinical symptoms of PD include a number of movement perturbations, including resting tremors, rigidity, akinesia (or bradykinesia), and postural instability. However, motor symptoms appear only after the loss of over 60% of dopaminergic neurons, accompanied by an 80–85% decrease in dopamine (DA) concentrations in the substantia nigra pars compacta (SNpc) [2]. Oxidative stress (OS) is one of the main mechanisms involved in the neurodegeneration of SNpc dopaminergic neurons in PD [3]. It is characterized by excessive formation of reactive oxygen species (ROS), accompanied by impaired function of the cellular antioxidant system. A primary source of ROS is impaired mitochondrial function. Both mitochondrial dysfunction and OS are accepted as key molecular mechanisms of neurodegeneration in PD [4].

Due to the high frequency of sporadic, non-genetic PD, estimated to be approximately 90% of all documented cases, it is assumed that the etiology of PD involves a variety of different factors [5]. According to epidemiological data, exposure to exogenous toxins such as pesticides, metals, and solvents can increase the risk of developing PD down the line. The interplay between both environmental and genetic factors appears to cause the majority of PD cases [1,6,7]. Rotenone, originally developed as a pesticide, is frequently used to model PD in laboratory conditions [8]. This compound inhibits complex I of the mitochondrial electron transport chain, causing OS, alpha-synuclein aggregation, and Lewy body formation, leading to the death of DA neurons [9]. The activation of glial cells and initiation of a neuroinflammatory response accompanying the initial neuron death exacerbates DA neuron loss [10,11].

Despite decades of research, there is still no cure for PD. Existing pharmacological therapies focus on replacement therapy, which aims to restore DA levels [12], and do not target the underlying pathological mechanisms. Carnosine (beta-alanine-L-histidine), a dipeptide, is a natural hydrophilic antioxidant with a low molecular weight, present in the muscle and nervous tissue of humans and other mammals [13]. Its administration prevents locomotor depression and muscle rigidity, as well as normalizes the brain’s antioxidant status in rodent models of parkinsonism [14,15,16].

Carnosine has been shown to alleviate symptoms in a wide array of parkinsonism models in rodents. In a 1-methyl-4-phenyl-1,2,3,6-tetrahydropyridine (MPTP)-induced parkinsonism model in SAMP1 (Senescence Accelerated Mice, Prone) mice, which are characterized by a chronically elevated tissue level of free radicals, the addition of carnosine (100 mg per kg of body weight) to drinking water accompanying MPTP administration over the course of 8 days prevented the development of a number of symptoms, including locomotor activity depression and muscle rigidity [14]. In a model of 6-OHDA-induced parkinsonism in Wistar rats, two intraperitoneal injections of carnosine (250 mg/kg) 1 day and 1 h prior to intrastriatal 6-OHDA injection significantly decreased the number of apomorphine-induced rotations (the test is commonly used for evaluating the development of parkinsonism in animal models), reduced apoptosis in the SNpc, and normalized malonic dialdehyde and nitrite concentrations along with catalase activity in the midbrain [15].

A pilot clinical study published in 2008 has demonstrated that the introduction of carnosine to the therapeutic regimen of PD patients significantly enhances the effect of therapy on neurological symptoms and correlates with improvements in the endogenous antioxidant status of patients [17]. Symptoms affected by carnosine administration included improved motor activity overall, alleviation of hypokinesia severity, rigidity, and tremor, as well as better performance in the “everyday activity” test compared with the basic therapy group. The observed increase in the effectiveness of PD therapy in response to the use of antioxidants demonstrates their applicability in such pathologies. However, the use of carnosine in therapy is limited by its susceptibility to hydrolysis by tissue and serum carnosinases, which decreases its bioavailability. This issue can be resolved by modifying carnosine in a way that will make the dipeptide itself resistant to hydrolysis or by binding it into a compound that precludes enzyme access.

Another promising lipophilic antioxidant is alpha-lipoic acid (ALA). ALA is a small molecule that has two oxidated or reduced (dihydrolipoic acid–DHLA) thiol groups. In vivo, DHLA is present in the mitochondria, where it is used as a coenzyme for the pyruvate dehydrogenase and alpha-ketoglutarate dehydrogenase complexes. Both DHLA and ALA are capable of directly scavenging ROS and regenerating endogenous antioxidants, including glutathione [18] and vitamins C and E [19]. Due to this combination of properties, ALA is viewed as a promising therapeutic agent [20]. The potential applicability of ALA in PD therapy is undergoing active research, both in vivo and in vitro. For example, ALA has been shown to alleviate a variety of symptoms in a model of unilateral intrastriatal injection of 6-OHDA in rats, including suppression of rotation in the apomorphine rotation test and increased performance of animals in various locomotor tests, including the open field test. These effects are mediated by the ALA regulating brain tissue catalase activity and impeding the accumulation of thiobarbituric acid (TBA)-reactive oxidation products and nitrites, in turn, suppressed peroxidation and facilitated neuron survival in the striatum [21].

However, ALA has poor solubility in water and low bioavailability (about 30%) due to its hepatic degradation and instability in the stomach [22]. To reduce the limitations of both ALA and carnosine and increase their therapeutic applicability, a new complex was developed—a water-soluble nanomicellar construct of carnosine with ALA (CLA nanomicellar complex, patent number RU2647435 from 15 March 2018). Lyophilized CLA is water-soluble and forms nanoparticles approximately 25 nm in diameter, comprised of micelles with a hydrophobic core (ALA) and hydrophilic shell (carnosine). Previously, our research group has shown the neuroprotective properties of CLA in a model of MPTP-induced parkinsonism in rats, where it normalized DA and serotonin (5-HT) metabolism and attenuated lipid peroxidation (LPO) [23].

The aim of this study was to evaluate the neuroprotective action of CLA in a model of late-stage (motor) parkinsonism induced via daily subcutaneous injections of rotenone over the course of 18 days. To assess the effectiveness of CLA, physiological characteristics, including weight, food intake, and locomotor activity in the open field test, were evaluated. Furthermore, we evaluated the antioxidant status of brain tissue, the level of monoamines and their metabolites in the striatum, and neuron density in the SNpc of animals.

## 2. Materials and Methods

### 2.1. Animals

Studies were carried out on 46 outbred Wistar male rats aged 2.5–3 months, weighing 230–330 g, kept in standard controlled vivarium conditions with a 12-h light/dark cycle. Prior to the beginning of the experiment, the animals had no restrictions on food intake and had free access to water throughout the entire duration of the study. All experiments were carried out in the daytime (from 9:00 to 20:00). The maintenance of animals and conduct of experiments were carried out in accordance with international rules and ethical standards. The study adheres to the principles delineated in the “European Convention for the Protection of Vertebrate Animals used for Experimental and other Scientific Purposes” (Strasbourg, 1986). The design of the study was approved by the local ethics committee of the Federal State Budget Scientific Institution Scientific Center for Scientific Research (Protocol No. 14-7/21 dated 10 July 2021).

### 2.2. Experimental Design

Systemic administration of the mitochondrial toxin rotenone was used as a model to observe the gradual slow development of parkinsonian symptoms in experimental animals with a pronounced premotor and motor stage [24]. To induce parkinsonism, animals received subcutaneous injections of 2 mg/kg of body weight rotenone dissolved in 2% DMSO and sterile vegetable oil over the course of 18 days (5 days of injections, 2 days off, regimen 5/2). Immediately prior to injection, the suspension was thoroughly mixed to ensure even distribution. Every day, simultaneously with the toxin, the animals received intraperitoneal injections of the studied compounds: CLA nanomicellar complex 50 or 25 mg/kg of body weight, carnosine 50 mg/kg of body weight, prepared in saline solution (pH 7.2–7.4), or saline solution, in accordance with the experimental protocol.

In accordance with the goals of this study, 5 animal groups were delineated in the experiment: 1. “Control” (subcutaneous injections of the solvent + intraperitoneal injections of saline in the volume corresponding to the studied preparations) (*n* = 9); 2. “Rotenone” (subcutaneous injections of rotenone solution + physiological saline intraperitoneally in the volume corresponding to the studied compounds) (n = 10); 3. “Carnosine” (subcutaneous injections of rotenone solution + intraperitoneal carnosine at a dose of 50 mg/kg of body weight) (n = 9); 4. “CLA 25” (subcutaneous injections of rotenone solution + intraperitoneal CLA complex at a dose of 25 mg/kg of body weight) (n = 9); 5. “CLA 50” (subcutaneous injections of rotenone solution + intraperitoneal CLA complex at a dose of 50 mg/kg of body weight) (n = 9).

In order to continuously monitor the condition of the animals in the experiment, the weight of the animals was recorded every 2 days. Moreover, on day 11, the amount of food consumed per hour was assessed. Animals were tested in an open field test (OF) to assess motor dysfunction on days 7 and 18. On the 18th day of the experiment, a parkinsonism-specific test for the strength of the forelimbs (Grip strength measurement), reflecting the presence of muscle rigidity in animals, was also performed.

Brain tissues were collected on the 19th day. Animals were decapitated 24 h after the last injection, the brain was removed on ice, and the frontal lobes and rostral regions of the striatum were isolated. The isolated areas of the brain were placed in test tubes and immediately frozen in liquid nitrogen. The brain block containing the SNpc was also frozen in liquid nitrogen vapor for further histological examination. Brain tissue samples were stored at −80 °C.

### 2.3. Open Field Test

This test was used to study the locomotor activity of rats. The experiments were carried out in a round open field with a diameter of 97 cm and a wall height of 42 cm (Open Science, Moscow, Russia). The floor of the apparatus was divided into 3 rows of equal-area sectors, which made it possible to determine the distance traveled by the number of sectors crossed. Behavior was recorded using the RealTimer program, v. from 31.01.2009 (Open Science, Moscow, Russia). The following parameters were evaluated over the course of 5 min: the distance traveled, the number of rearing instances and the duration of “freezing” episodes.

### 2.4. Grip Strength Test

The grip strength of the forelimbs was measured using a digital grip force meter on day 18 of the experiment after the last injection of rotenone. The rat was positioned to grasp the grid with its forelimbs, after which the grid was gently pulled to record grip strength [25]. Each experiment consisted of three trials, after which the mean value was calculated in kilogram-force (Kgf).

### 2.5. Histological Detection of Cells by Nissl Staining and Morphometric Assessment

On a Leica CM1950 cryostat (Leica, Wetzlar, Germany), a series of coronal sections 20 µm thick was made from each brain block and placed on glass slides (Thermo Scientific, Waltham, MA, USA). Sections were taken under visual control, referring to the stereotaxic atlas of the rat brain [26] at a distance of −4.92 to −5.4 mm from Bregma to ensure the presence of the substantia nigra pars compacta (SNpc) on the resulting slices. Slides with sliced brain tissue were stored at −70 °C.

Sections were fixed in cold 4% paraformaldehyde solution (Sigma, MO, USA) for 15 min. After fixation, sections were incubated 3 times for 5 min in 1× PBS. They were then placed in a solution of toluidine blue (Labiko, Saint Petersburg, Russia) for 10 min at a temperature of 25 °C for staining according to the Nissl method. After staining, the sections were washed from the dye 3 times for 5 min in 1 × PBS. Then, they were rinsed in 96% ethanol and differentiated in 96% ethanol for 3 min. Dehydration was carried out in 99% isopropanol (BioVitrum, Saint Petersburg, Russia) for 2 min, after which it was clarified twice in ortho-xylene for 1.5 min and placed in a synthetic BioMount cover medium (Bio-Optica, Milano, Italy) under a coverslip (Thermo Scientific, MA, USA).

Sections were photographed using a Nikon Eclipse E200 (Nikon, Tokyo, Japan) light microscope at 4× magnification using the NIS-Elements software (Nikon, Tokyo, Japan). Stained cells in the SNpc were counted using the ImageJ program (National Institutes of Health, Bethesda, MD, USA). On each photograph, the borders of SNpc were manually traced in accordance with the rat brain atlas, after which the cells in the selected area were manually counted. Cell number analysis was performed separately for the right and left hemispheres on each slice. The data were averaged for the hemispheres and for two slices from each brain.

### 2.6. Measurement of the Content of Monoamine Mediators and Their Metabolites

The content of monoamines was determined in the striatum of experimental animals using high-performance liquid chromatography with electrochemical detection (HPLC/ECD) on an LC-304T chromatograph (BAS, West Lafayette, IN, USA) with a Phenomenex analytical column (C18, 4 × 150 mm, 4 mcM) in accordance with the Yang protocol [27]. Before the experiment, brain samples were homogenized (Teflon-glass) in 20 volumes of 0.1 N HClO_4_ with the addition of dioxybenzylamine (0.5 nmol/mL) as an internal standard for the determination of monoamines (dioxybenzylamine is a catecholamine substance that is not found in native tissue). Samples were centrifuged at 10,000× *g* for 15 min; the supernatant was used to evaluate monoamine presence. The content of the following monoamines and products of their metabolism was determined: serotonin (5-HT), 5-hydroxyindoleacetic acid (HIAA), noradrenaline (NA), dopamine (DA), dihydroxyphenylacetic acid (DOPA), 3-methoxytyramine (3-MT), homovanillic acid (HVA).

### 2.7. Measuring Iron-Induced Chemiluminescence in Biological Samples

LPO in animal brain tissue and the total antioxidant activity of the compounds were studied using the method of Fe^2+^ iron-induced chemiluminescence (CL) of biological samples (homogenates of brain tissue) proposed by Y.A. Vladimirov and adapted to the Luminometer-1251 (LKB, Sweden) [28,29]. The following parameters were analyzed: a rapid CL flash (h, mV), the intensity of which characterizes the level of pre-formed LPO products (mainly lipid hydroperoxides); latent period (τ, s), indicating the resistance of the substrate to further oxidation and reflecting the endogenous antioxidant potential; the maximum possible intensity of CL (H, mV), associated with the further oxidation of ferrous ions and the accumulation of LPO products.

Tissue samples (brain sections from animals) were homogenized in the presence of phosphate buffer (PB, 60 mM KH2PO4 and 105 mM KCl at pH 7.45). Then, 800 µL of PB was added to 100 µL of 10% tissue homogenate in the measuring cuvette. The cuvette with the resulting suspension was placed in the measuring chamber of the Luminometer-1251 (LKB, Sweden) (heated to a temperature of 37 °C and stirred constantly), and the background values were recorded. To initiate the CL reaction, 0.1 mL of ferrous iron solution (FeSO_4_·7H_2_O) at a final concentration of 2.5 mM was added to the cuvette using a dispenser, and the luminescence curve was recorded.

### 2.8. Statistical Analysis

Statistical data processing was performed using Statistica 10.0 and GraphPad Prism 8.0 software. To determine the significance of differences, factorial analysis of variance (one-way ANOVA) was used with further post hoc analysis using Tukey’s or Bonferroni’s test for multiple comparisons (one-way ANOVA followed by Tukey’s, Bonferroni or Dunn’s post hoc test for multiple comparisons was used). Differences were considered significant at *p* < 0.05. Results are presented as mean ± standard error of the mean (M ± SEM).

## 3. Results

### 3.1. Weight and Food Consumption Rate

**Figure 1:**
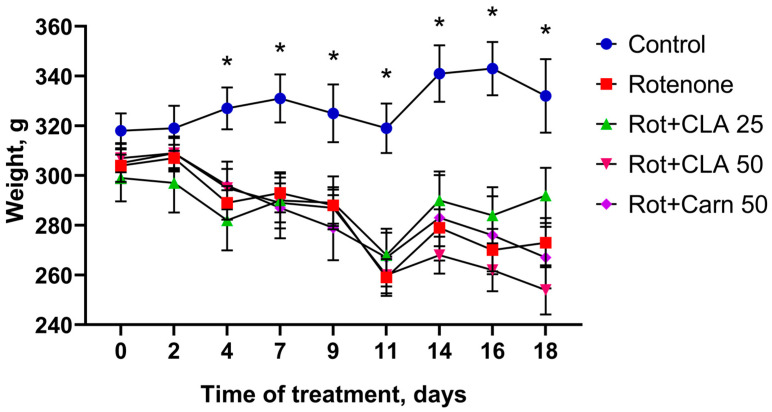
Dynamics of animal weight loss over the course of 18 days, weight was measured every 2 days. *—difference from intact animals, *p* < 0.05.

The weight of intact animals did not fluctuate significantly over the course of the 18 days of the experiment, while all animals treated with rotenone lost weight. Significant weight loss (Figure 1) in animals belonging to the “Rotenone” group was observed starting from day 4 of toxin administration (289.9 ± 6.7 g vs. 327.4 ± 8.4 g in control, *p* < 0.01) and continued in subsequent days. On day 11, a peak in weight loss was observed (259.8 ± 7.4 g versus 319 ± 9.9 g in control, *p* < 0.001), which preceded the peak of mortality observed on day 12–11.1% of the total number of animals.

Treatment with CLA did not attenuate the weight loss observed in the “rotenone” group on all days of the experiment. However, by day 18, the “CLA 25” group lost the least weight (292.6 ± 11.1 g versus 332.0 ± 14.7 g in the control, *p* = 0.05), while the group “CLA 50” lost the most (254.0 ± 9.9 g, *p* < 0.001 from control and *p* < 0.01 from “CLA 25”).

One-way ANOVA showed a significant effect of rotenone administration on food intake (F (4, 36) = 3.519, *p* < 0.01) (see Supplementary Materials, Table S1). On day 3 of the experiment, a trend towards a decrease in the amount of food consumed by animals in the rotenone group was observed (3.3 ± 0.6 g vs. 5.4 ± 0.7 g in the control, *p* < 0.07), and it decreased significantly both relative to the control (2.7 ± 0.4 g vs. 4.2 ± 0.5 g *p* < 0.05) and relative to day 0. The amount of food consumed by animals treated with carnosine in addition to rotenone also significantly decreased (to 2.4 ± 0.5 g, *p* < 0.05). The introduction of either of the two CLA doses to rotenone-injected animals tended to increase the amount of food consumed on the eleventh day of the experiment (up to 3.3 ± 0.4 g at a dose of 25 mg/kg and 3.2 ± 0.8 g at a dose of 50 mg/kg), but the differences remained insignificant relative to the “rotenone” group and did not differ significantly from intact animals.

As such, administration of the studied compounds in conjunction with rotenone did not affect animal weight. The most significant weight loss was observed among animals belonging to the “CLA 50” group, which may be due to a CLA overdose.

### 3.2. Locomotor Activity in the Open Field Test

Starting from day 4, rotenone-treated animals developed postural instability, reflected in an arched posture while traversing the open field. Analysis of variance showed that on day 7, rotenone administration caused a significant increase in the duration of freezing episodes (F (4, 43) = 3.027, *p* = 0.0276) and a decrease in distance travelled in the OF test (F (4, 43) = 6.977, *p* = 0.0002), but had no effect on rearing behavior (used as a measure of exploratory activity) (F (4,43) = 0.8069, *p* = 0.0652). On day 18, rotenone administration caused a further significant increase in freezing duration (F (4, 36) = 4.115, *p* = 0.008) and a decrease in rearing behavior (F (4, 36) = 6.055, *p* = 0.001), while changes in locomotor activity were insignificant (F (4, 36) = 2.246, *p* = 0.0868).

**Figure 2:**
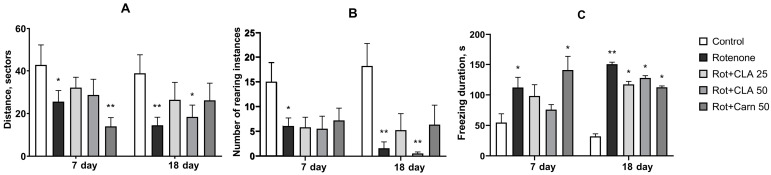
Changes in motor activity on 7 and 18 days of experiment. (**A**) Locomotor activity; (**B**) rearing behavior; (**C**) freezing duration, sec. *—difference from intact animals with *p* < 0.05; **—difference from intact animals with *p* < 0.01 by Kruskal-Wallis test with Dunn’s correction for multiple comparisons.

Post-hoc analysis showed significant differences between the “Rotenone” group and intact animals in all studied parameters (Figure 2). On day 7, locomotor activity (Figure 2A) was significantly reduced relative to intact animals in the “Rotenone” (*p* ≤ 0.05) and “Carnosine” (*p* < 0.01) groups (25.6 ± 5.2 sectors and 14.0 ± 4.1 sectors, respectively, versus 42.8 ± 9.4 in intact animals). The locomotor activity of animals receiving CLA at both concentrations was increased relative to animals receiving only rotenone (32.1 ± 4.8 and 28.7 ± 7.3 squares, respectively) and did not significantly differ from intact animals. On day 18, in the “Rotenone” group, this parameter decreased even further—to 14.5 ± 3.8 sectors against 38.9 ± 8.6 sectors in the control (*p* = 0.01), and it also decreased in the “CLA 50“ group −18.4 ± 5.5 sectors. The locomotor activity of animals in the “CLA 25” group remained at approximately the same level as on day 7 (26.4 ± 8.1 squares), while in the “Carnosine” group, it increased (26.2 ± 8.0 sectors), and for both groups did not significantly differ either from control animals or from animals that received only rotenone.

Rearing behavior (Figure 2B) on day 7 was significantly reduced relative to control animals only in the “Rotenone” group (6.1 ± 1.6 rears versus 15.0 ± 3.9 in intact animals, *p* = 0.028). In animals receiving the investigated compounds, it was also reduced (in the “Carnosine” group 7.2 ± 2.5; “CLA 25” 5.9 ± 2.0; “CLA 50” 5.6 ± 2.5 rears) but did not differ significantly from intact animals or from the “rotenone” group due to high variability in the acquired values. For example, one of the animals in the “CLA 25” group was not highly active and did not display any rearing behavior whatsoever—in the meantime, a different animal from the same group performed 15 rears over the course of the 5 min spent in the open field. In the “Rotenone” group, four of the animals remained inactive. On day 18, the rearing behavior of animals in the “Rotenone” group decreased even further relative to the intact animals (1.6 ± 1.2 rears versus 18.2 ± 4.5 in the intact animals, *p* < 0.01). The number of rears in animals treated with the studied compounds also remained reduced and did not differ significantly from animals in the “Rotenone” group. However, the number of rears in animals in the “Carnosine” group (6.4 ± 3.9) decreased to a lesser extent relative to day 7 and did not differ significantly from intact animals. In animals of the “CLA 25” group, rearing behavior also decreased to a lesser extent relative to day 7 (up to 5.3 ± 3.3 rears). It should be noted that in the “Rotenone” group, seven animals did not rear at all, while in the “CLA 25” and “CLA 50” groups, only one and four animals, respectively, did not display any rearing behavior. In the “Carnosine” group, only two animals lacked rearing activity.

On day 7, the duration of freezing episodes (Figure 2C) in the “Rotenone” group was 112.2 ± 16.7 s versus 54.7 ± 14.3 s in the control (*p* = 0.032), and by day 18, it increased even further—150.5 ± 69.3 s versus 32.0 ± 4.1 s in control (*p* < 0.001). The administration of carnosine and CLA did not significantly affect this parameter; however, on day 7, the freezing duration in the “CLA 50” group (8.2 ± 2.2 s) did not significantly differ from both the intact animals or the “Rotenone” group. On day 18, this parameter was reduced relative to the “Rotenone” group in animals of all groups receiving the investigated compounds, and the significance of differences from control animals was *p* = 0.01, while the “Rotenone” group differed from intact ones with *p* < 0.001.

### 3.3. Forelimb Grip Strength Measurement Test

**Figure 3:**
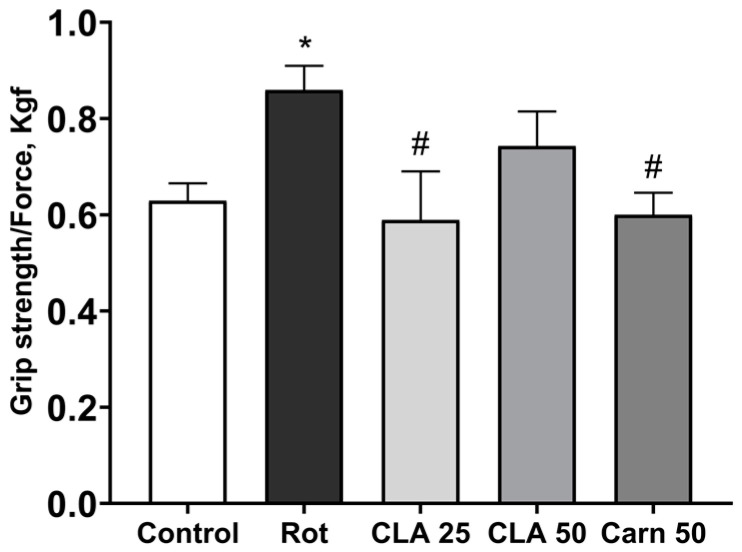
Evaluation of animal strength in the grip strength measurement test. *—*p* < 0.05 compared to the control group. #—*p* < 0.05 compared to the “rotenone” group in univariate analysis using Dunnett’s correction for multiple comparisons.

The grip test was performed to evaluate parkinsonism symptoms. Increase grip strength in rotenone treated animals may be the result of forelimb rigidity. It was shown that intact and experimental animals differed in grip strength (F (4, 36) = 3.681, *p* = 0.0149), recorded in Kgf. On day 18, rats treated with rotenone held onto the grid more strongly than the control animals (0.86 ± 0.05 Kgf versus 0.63 ± 0.04 Kgf in the control, *p* = 0.027) (Figure 3). Grid retention decreased relative to the “Rotenone” group in animals treated daily with 25 mg/kg CLA (to 0.59 ± 0.1 Kgf, *p* = 0.016) and 50 mg/kg carnosine (0.6 ± 0.05 Kgf, *p* = 0.031) and did not differ from intact animals.

### 3.4. Density of Neurons in the SNpc

**Figure 4:**
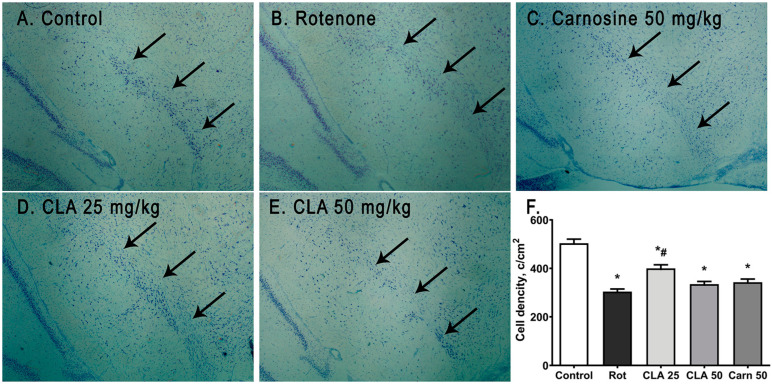
Photographs of neurons stained according to the Nissl method in the SNpc (marked with arrows), brain section: (**A**) intact animal; (**B**) animals who received subcutaneous injections of rotenone; (**C**) animals who received subcutaneous injections of rotenone and intraperitoneal injections of the CLA complex at a dose of 25 mg/kg of body weight; (**D**) animals who received subcutaneous injections of rotenone and intraperitoneal injections of the CLA complex at a dose of 50 mg/kg of body weight; (**E**) animals who received subcutaneous injections of rotenone and intraperitoneal injections of carnosine at a dose of 50 mg/kg of body weight; (**F**) neuron density count data in the SNpc, stained according to the Nissl method. *—differences from intact animals with *p* < 0.0001; #—differences from animals that received rotenone with *p* = 0.0001.

The distribution density of neurons in the SNpc was determined via histochemical staining according to Nissl (Figure 4A). One-way ANOVA showed a significant effect of rotenone administration on neuronal density (number of neurons/area of SNpc, Figure 4F) in SNpc (ANOVA F (4, 36) = 35.29, *p* < 0.0001).

Further post hoc analysis of neuronal distribution density revealed its significant decrease by 39.6% in the “Rotenone” group relative to intact animals (*p* < 0.0001). At the same time, the introduction of the CLA complex at a dose of 25 mg/kg of body weight increased this parameter by 19% (*p* = 0.0001), which indicates a direct neuroprotective effect of the CLA complex on SNpc neurons. While in animals that received only rotenone, the density of neurons in the SNpc averaged 304.0 ± 10.8, in the “CLA 25” group, it averaged 399.8 ± 15.0. At the same time, in intact animals, the distribution density of neurons in the SNpc constituted 503.7 ± 17.0.

### 3.5. The Content of Monoamine Mediators and Their Metabolites in the Striatum

**Figure 5:**
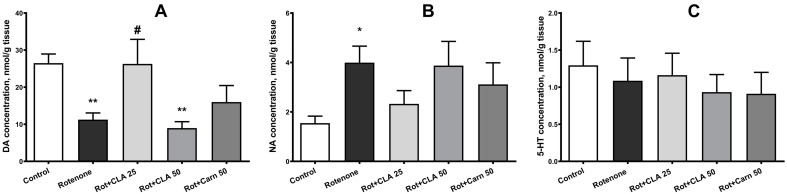
Effect of subcutaneous administration of rotenone, accompanied by the administration of carnosine (50 mg/kg) or carnosine-lipoic acid (CLA) complex (CLA) (25 and 50 mg/kg) for 18 days on the content of neurotransmitters: (**A**) dopamine (DA), (**B**) noradrenaline (NA), (**C**) serotonin (5-HT) in the striatum of experimental animals. *—differences from intact animals with *p* < 0.05, **—*p* = 0.01; #—differences from animals that received rotenone with *p* < 0.05.

The HPLC/ECD method showed that both rotenone and the studied compounds affect striatal DA levels (ANOVA F (4, 36) = 5.46, *p* = 0.0022; Figure 5A), while noradrenaline (NA) (*p* = 0.0727, Kruskal-Wallis test; Figure 5B) and serotonin (5-HT) (ANOVA F (4, 36) = 0.2621, *p* = 0.8995; Figure 5C) concentrations did not change significantly. Bonferroni`s post hoc testing revealed a 57.6% decrease in striatal DA concentrations of rotenone-treated rats (11.2 ± 1.8 nmol/g of tissue versus 26.5 ± 2.5 nmol/g of tissue in control animals, *p* = 0.01). Rotenone administration also caused a 2.58-fold increase in NA content– (4.0 ± 0.7 nmol/g of tissue versus 1.5 ± 0.3 nmol/g of tissue in control animals, *p* = 0.0347, Dunn’s test).

CLA at a dose of 25 mg/kg of body weight caused a significant increase in DA levels to 23.7 ± 6.1 nmol/g of tissue (2.2 times compared to the “Rotenone” group levels on day 18, *p* = 0.0456), which corresponds to changes in SNpc neuron density (Figure 5F). While 25 mg/kg CLA appears to cause a slight decrease in NA levels, it does not significantly differ from intact animals or from the “Rotenone” group.

In all experimental groups, no changes in the content of the main DA metabolites-3,4-dihydroxyphenylacetic acid (DOPAC), homovanillic acid (HVA) and 3-methoxytyramine (3-MT), as well as the 5-HT metabolite 5-hydroxy indoleacetic acid (HIAA) according to the analysis of variance were observed (see Supplementary Materials, Figures S1–S4). However, post hoc analysis revealed a 62.8% decrease in 3-MT concentration (0.6 ± 0.1 nmol/g tissue vs. 1.6 ± 0.3 nmol/g tissue in control animals, *p* = 0.02, Dunn’s test, Figure S2). In all three groups receiving a compound in conjunction with rotenone, none of the investigated metabolites differed significantly from either the “Rotenone” or control groups.

### 3.6. Antioxidant Status of the Brain

**Figure 6:**
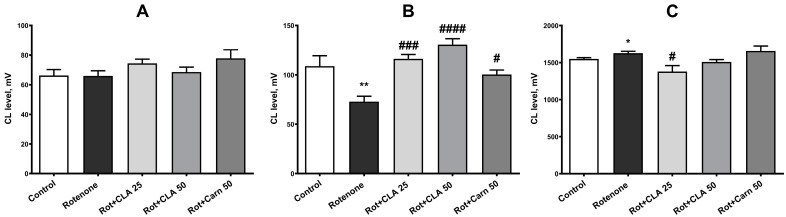
Effect of subcutaneous administration of rotenone for 18 days, as well as joint administration of carnosine (50 mg/kg) and carnosine-lipoic acid complex (CLA) (25 and 50 mg/kg) with rotenone on: (**A**) the level of preformed hydroperoxides (h); (**B**) the total activity of the antioxidant system (t) and (**C**) the maximum intensity of CL (H), mV in the homogenates of the frontal lobes of experimental animals. *—differences from intact ones with *p* < 0.05, **—*p* < 0.01; #—differences from animals that received rotenone with *p* < 0.05, ###—*p* <0.001, ####—*p* <0.0001.

The oxidative status in tissue homogenates of the frontal lobe of the cerebral hemispheres was assessed by CL and characterized using three kinetic parameters. Eighteen days of rotenone administration significantly affected total antioxidant activity (latent period of CL, parameter t, mV, Figure 6B) and the maximum intensity of tissue oxidation (parameter H, mV, Figure 6C) (F (4,36) = 11.98, *p* < 0.0001, and F (4,36) = 4.676, *p* = 0.0051, respectively). The level of preformed lipid hydroperoxides (parameter h, mV, Figure 6A) remained within the control values for all groups (F (4, 36) = 1.686, *p* = 0.1793). Post-hoc analysis revealed that rotenone administration caused a 33.0 ± 7.6% decrease in the total antioxidant activity of brain tissue (72.9 ± 5.5 mV versus 108.8 ± 10.5 mV in control, *p* = 0.0024), and there was also an increase maximum intensity of oxidation by 5.6 ± 1.6% (1627 ± 25 mV versus 1550 ± 17 mV in control, *p* = 0.03).

All studied compounds significantly increased the total antioxidant activity of the frontal lobe tissue: carnosine by 25.3 ± 4.2% (*p* = 0.036), “CLA 25” by 39.7 ± 4.1% (*p* = 0.0002), “CLA 50“—by 53.1 ± 5.5% (*p* < 0.0001). At the same time, in the “CLA 25” group, this parameter corresponded to control animals, and in the “CLA 50“ group exceeded it. The maximum intensity of LPO was reduced only by the introduction of CLA at a dose of 25 mg/kg (by 15.9 ± 5.1%, *p* = 0.0008 relative to animals receiving only rotenone).

Thus, we have shown a decrease in the latent period of CL, which reflects a decrease in the endogenous antioxidant potential of the tissue, and an increase in the maximum intensity of CL on day 18, indicating the depletion of the antioxidant system and activation of LPO processes. The introduction of carnosine and the CLA complex throughout the entire period, starting from the first day of animals receiving rotenone, prevents the development of OS in the brain tissue.

## 4. Discussion

In recent decades, there has been an increase in the prevalence of neurodegenerative diseases. One such disease is Parkinson’s disease (PD), characterized by progressive degeneration of dopaminergic neurons in the substantia nigra pars compacta (SNpc) [30]. While some researchers believe that the main factors involved in the development of PD are population aging and genetic mutations, a number of studies have shown that PD can be caused by exposure to certain exogenous toxic substances, which can cause high levels of oxidative stress [31]. In light of this information, the need for antioxidant drugs in PD therapy becomes evident.

To study the neuroprotective efficacy of the antioxidant complex CLA, we induced the motor stage of PD in rats via chronic subcutaneous administration of 2 mg/kg body weight of the mitochondrial toxin rotenone (daily for 18 days). Daily chronic systemic administration of rotenone, which is liposoluble and penetrates the blood-brain barrier, causes slow progression of PD in animals. However, although this model is convenient, it can cause a wide variety of peripheral side effects and high mortality rates in animals depending on the dose used. Previously, Zhang ZN et al. showed that daily subcutaneous injection of 2 mg/kg rotenone promoted the development of parkinsonism symptoms in rats while maintaining relatively low mortality rates. Motor symptoms were accompanied by a significant decrease in the amount of tyrosine hydroxylase in the SNpc, decreased intrastriatal DA, and formation of alpha-synuclein aggregates in brain tissue [24]. As such, we used this dose in the present study. The mortality rate of the animals utilized in this study reached 16.3% by day 18 of the experimental procedures. 

In this model, the presence of two distinct stages of pathology development was demonstrated. The first stage is characterized by the disruption of motivational, emotional, and cognitive components of behavior and occurs over the course of the first 3 days of injection. From day 4 of toxin administration, the motor stage begins, characterized by a significant decrease in the SNpc neuron density and changes in dopaminergic system function, including a decrease in dopamine (DA) levels in the striatum, bradykinesia, rigidity, postural instability, and a decrease in locomotor activity [32]. In our study, motor deficits appeared by day 4 of rotenone administration, which worsened by the end of the experiment. In addition to hypokinesia, animals which received subcutaneous injections of rotenone were subject to the development of forelimb rigidity by the end of the experiment. Previously, in similar models of chronic rotenone-induced PD (2 mg/kg), it was shown that muscle rigidity and bradykinesia could be observed on day 7 [33] of rotenone injection. In sum, animals experienced muscle rigidity from day 4 until the end of the toxin administration. As such, we conducted our evaluation of the neuroprotective efficacy of the CLA complex in a model of PD, which reproduces the development of the main motor symptoms of the disease, namely, hypokinesia and muscle rigidity.

At doses of 50 or 25 mg/kg, the CLA nanomicellar complex did not lead to a significant improvement in the physiological parameters of the experimental animals. The introduction of 25 mg/kg CLA partially restored values in the grip strength test to control levels, which can be interpreted as a decrease in muscle rigidity. At the same time, in the open field test, an insignificant increase in spontaneous locomotor activity and a decrease in freezing duration on days 7 and 18 were observed, while the number of rearing instances remained unchanged relative to the “Rotenone group” on day 7 and increased insignificantly on day 18 only in animals treated with 25 mg/kg CLA. Results acquired by other authors on MPTP-induced PD models indicate that the exploratory activity of animals in the open field test is a highly sensitive parameter of motor deficit and correlates with striatal DA content [34].

Weight monitoring is frequently used in studies to assess overall animal health [35,36]. A significant decrease in the weight of animals in the experimental group was observed starting from day 4 and correlated with decreased locomotor activity. Weight loss may be explained by a gradual decrease in the amount of food consumed due to decreased food motivation and motor impairment. Earlier studies of emotional and motivational disturbances in rat behavior after chronic rotenone administration showed a decrease in foraging behavior and the development of anhedonia before the development of motor disorders, which may be the primary reason for the decrease in food intake which we observed [32]. Impaired motivation develops as a consequence of SNpc damage and decreased levels of DA and its metabolites after rotenone administration [37], which were observed in the present study. Animal studies have shown that LA supplementation helps reduce body weight and fat mass by reducing food intake, increasing energy expenditure, and improving skeletal muscle energy metabolism [38,39]. As such, the observed significant weight loss in the 50 mg/kg CLA group may be a direct consequence of a high LA content. The group of animals which received 25 mg/kg CLA in addition to rotenone, meanwhile, lost the least weight by the end of the experiment.

Histochemical staining of neurons in SNpc and the content of monoamine mediators and their metabolites in the striatum of experimental animals was carried out to assess neurodegenerative changes. While the most commonly used method for quantitative analysis of dopaminergic neurons in SNpc is immunohistochemical staining for tyrosine hydroxylase [24,40], some studies indicate that manual counting of Nissl-stained SNpc cell number gives similar values to the number of tyrosine hydroxylase (TH)-positive neurons in this area [41,42]. In our study, we observed a 39.6% decrease SNpc neuron number, accompanied by a decrease in striatal DA levels by 57.6% in rats treated with only rotenone. In a previous study, subcutaneous administration of 2 mg/kg rotenone for 5 weeks resulted in a comparable loss of 41.9% of TH-positive neurons in SNpc and a 45.3% decrease in striatal DA content [24]. Administration of the CLA complex in conjunction with rotenone increased the neuron distribution density in the SNpc by 19% and striatal DA content by 56.8% compared with the stand-alone rotenone-treated animals. In another study, prolonged rotenone administration (35 days) led to a 25% decrease in the number of TH-positive neurons, which was ameliorated by administration of 100 mg/kg polyamine agmatine prior to rotenone injection (23.2% increase in TH-positive neurons relative to rotenone-treated animals) [43]. In a similar model of PD induced via six subcutaneous injections of 1.5 mg/kg of rotenone every 48 h, peroral administration of 50 mg/kg of LA led to an increase in the number of TH-positive and Nissl stained neurons, as well as an increase in striatal DA levels. The administration of 100 mg/kg L-carnitine simultaneously with LA increased these parameters as well [42].

In addition, we observed a decrease in the content of the DA metabolite 3-MT by 62.8% and an increase in the level of intrastriatal noradrenaline (NA) by 2.58 times in animals that received only rotenone. The observed decrease in intrastriatal 3-MT concentration may be due to decreased catechol-*O*-methyl transferase (COMT) activity or an increase in the activity of monoamine oxidase (MAO), which, together with aldehyde dehydrogenase, is involved in the reduction of 3-MT to HVA [44]. The increase in NA can also be explained by activation of the DA-to-NA metabolism pathway mediated by DA-beta-hydroxylase or by a compensatory response to damage to NAergic neurons. The concentrations of 3-MT and NA in the striatum of animals that received the studied antioxidants in addition to rotenone did not differ from either intact animals or from animals that received only rotenone. We found no change in the levels of the major DA metabolites DOPAC and HVA on day 18. Troshev et al. showed that DOPAC and HVA levels significantly increased only on the fifth day of rotenone administration, and preceded a decrease in DA level, then returned to normal [32], which may be associated with a compensatory reaction to a decrease in DA as a result of the loss of SNpc neurons at an early stage of PD [45].

We also observed disruptions in the oxidative status under the action of rotenone, accompanied by a decrease in the total antioxidant activity and an increase in the maximum intensity of oxidation of the tissue of the frontal lobe of the cerebral hemispheres, which were corrected by the introduction of all the studied compounds. At the same time, antioxidant activity increased in proportion to CLA complex dose. Andreeva-Gateva P. et al. showed that administration of 35 mg/kg LA in 6-hydroxydopamine unilateral intrastriatal injected rats for 14 days led to a significant increase in glutathione peroxidase activity and a decrease in thiobarbituric acid-reactive substances [46]. At the same time, it is indicated that doses of 50–100 mg/kg are most often used in studies [21,40,42]. The therapeutic dose of carnosine also starts at 50 mg/kg and reaches 500 mg/kg with a single application; for example, in the work of Afshin-Majd S. et al. in the acute model of PD, unilateral intrastriatal 6-OHDA-lesioned rats received intraperitoneal carnosine at a dose of 250 mg/kg twice, pre-surgery [15]. However, carnosine is frequently administered to animals with drinking water or intranasally in order to reduce its hydrolysis by carnosinases [47,48]. In our study, it was shown that the use of a complex of carnosine with ALA makes it possible to reduce the effective dose to 25 mg/kg and possibly even lower. This effect is achieved by improving the solubility of ALA and reducing the hydrolysis of carnosine – which undergoes hydrolysis of the CLA nanomicellar complex by serum carnosinase 20% slower than molecular carnosine.

Thus, the mild effect of the studied compounds on animal activity can be associated with high variability in parameter values in the experimental groups, presumably due to the different receptiveness of animals to the studied compounds and rotenone. It should also be noted that the studied compounds were administered simultaneously with the toxin, without preconditioning, which is usually done in studies evaluating antioxidants in order to enhance the protective effects. For example, daily administration of fucoidan, a sulfated polysaccharide derived from Laminaria japonica, was used 10 days prior to the start of rotenone administration and then throughout the entire time of subcutaneous administration of rotenone over the course of 4 weeks, which alleviated motor impairments and nigrostriatal dopaminergic degeneration in rats [49]. Two weeks pre- and post-treatment with the plant-derived antioxidant curcumin also demonstrated neuroprotective effects in the 6-hydroxydopamine-induced rat model of Parkinson’s disease, specifically by increasing the density of TH-positive neurons in the striatum and SNpc of animals [50].

The goal of our study was to investigate the therapeutic effect of the CLA complex on the progression of an existing pathological condition rather than as a prophylactic. In addition, the resulting insufficient effect of the CLA complex on the physiological characteristics of animals may be due to the doses chosen for the experiments being too high since 50 mg/kg had practically no effect on any of the studied parameters. Improving the bioavailability of these antioxidants seems to require a reduction in the effective dose in order to prevent excessive activity, which is reflected both in increased metabolism and associated weight loss and, potentially, in excessive antiradical activity, which reduces the level of reactive oxygen species below the minimum required for cell signaling [51]. Thus, in order to achieve a stable neuroprotective effect and symptomatic treatment, the use of lower doses of CLA and the use of L-DOPA, in addition to antioxidant therapy, may be beneficial.

## 5. Conclusions

The nanomicellar complex CLA combines the properties of two powerful natural antioxidants and improves their bioavailability. To summarize, in this study, we demonstrated that the administration of 25 mg/kg CLA ameliorates oxidative stress and physiological and neurochemical lesions in a model of PD induced by chronic (18 days) subcutaneous administration of rotenone. We found a 19% increase in the neuronal density in the SNpc with a simultaneous increase in DA levels in the striatum and an increase in the total antioxidant capacity of brain tissue compared to the toxin-only-treated group of animals. The neuroprotective effect of CLA was also reflected in the reduction of muscle rigidity in animals and an increase in their activity in the open field test. This study opens up the possibility of further studying the CLA complex as a potential antioxidant and neuroprotective agent, which may be beneficial in addition to L-DOPA therapy in Parkinson’s disease.

## Data Availability

The data presented in this study are available on request from the corresponding author. The data are not publicly available due to legal issues.

## Supplementary Materials

The following supporting information can be downloaded at: https://www.mdpi.com/article/10.3390/antiox12061215/s1, Table S1: Food consumed per hour; Figure S1: Effect of subcutaneous administration of rotenone, accompanied by the administration of carnosine (50 mg/kg) or carnosine-lipoic acid (CLA) complex (CLA) (25 and 50 mg/kg) for 18 days on the content of DA metabolites: A—3,4-dihydroxyphenylacetic acid (DOPAC), B—homovanillic acid (HVA) in the striatum of experimental animals; Figure S2: Effect of subcutaneous administration of rotenone, accompanied by the administration of carnosine (50 mg/kg) or carnosine-lipoic acid (CLA) complex (CLA) (25 and 50 mg/kg) for 18 days on the content of DA metabolite 3-methoxytyramine (3-MT); Figure S3: Effect of subcutaneous administration of rotenone, accompanied by the administration of carnosine (50 mg/kg) or carnosine-lipoic acid (CLA) complex (CLA) (25 and 50 mg/kg) for 18 days on the ratios metabolite/dopamine: A—DOPAC/DA, B—HVA/DA in the striatum of experimental animals; Figure S4: Effect of subcutaneous administration of rotenone, accompanied by the administration of carnosine (50 mg/kg) or carnosine-lipoic acid (CLA) complex (CLA) (25 and 50 mg/kg) for 18 days on the content of 5-HT metabolite 5-hydroxy indoleacetic acid (HIAA).
