# Supplementary material for: Neuroprotective Efficacy of a Nanomicellar Complex of Carnosine and Lipoic Acid in a Rat Model of Rotenone-Induced Parkinson’s Disease
Source: Antioxidants (Basel). 2023 Jun 4;12(6):1215. doi: 10.3390/antiox12061215 (PMC10294933; doi:10.3390/antiox12061215)
Supplement: Supplementary file 1 [file antioxidants-12-01215-s001.zip › antioxidants-2415844-supplementary.pdf]

Table S1. Food consumed per hour

	Intact	Rotenone	CLA 25	CLA 50	Carn 50
Mean	4,25	2,667	3,286	3,2	2,429
Std. Error of Mean	0,559	0,441	0,3595	0,8	0,4809

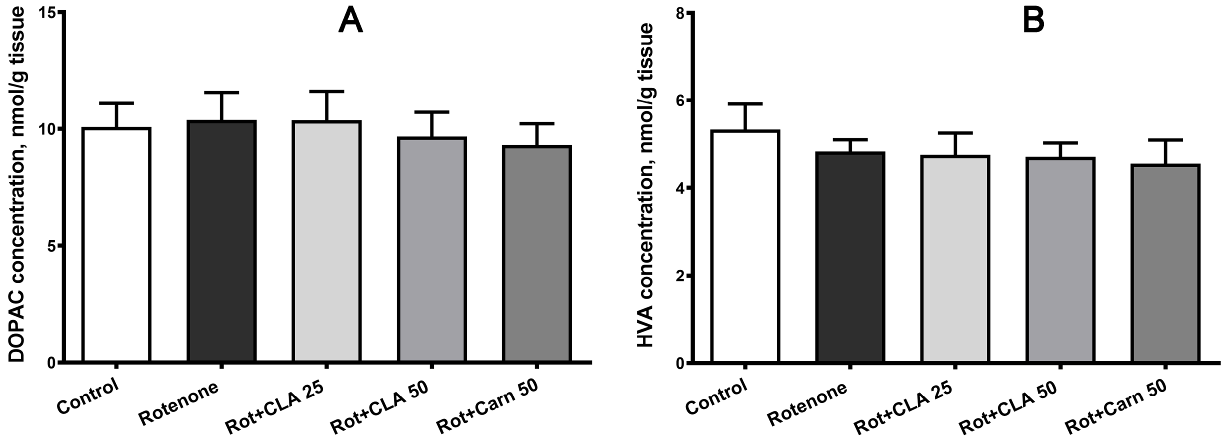

Figure S1. Effect of subcutaneous administration of rotenone, accompanied by the administration of carnosine (50 mg/kg) or carnosine-lipoic acid (CLA) complex (CLA) (25 and 50 mg/kg) for 18 days on the content of DA metabolites: A - 3,4-dihydroxyphenylacetic acid (DOPAC), B – homovanillic acid (HVA) in the striatum of experimental animals. * – differences from intact animals with $p < 0.05$, ** - $p = 0.01$; # - differences from animals that received rotenone with $p < 0.05$.

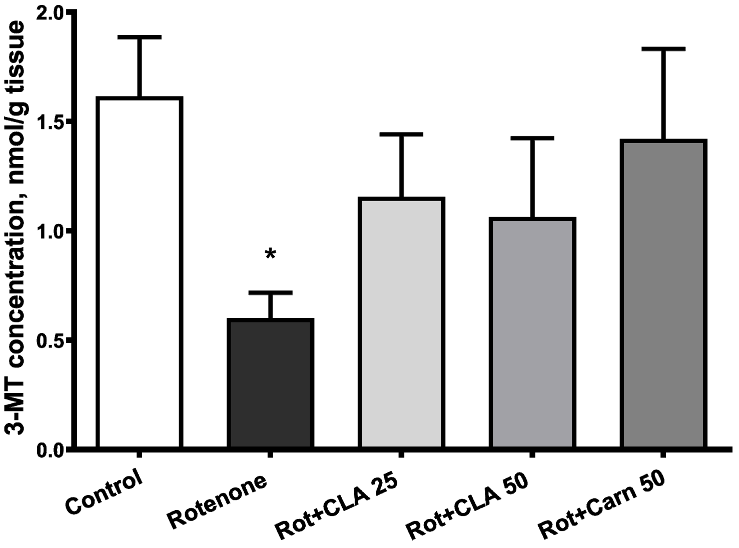

Figure S2. Effect of subcutaneous administration of rotenone, accompanied by the administration of carnosine (50 mg/kg) or carnosine-lipoic acid (CLA) complex (CLA) (25 and 50 mg/kg) for 18 days on the content of DA metabolite 3-methoxytyramine (3-MT). * – differences from intact animals with $p < 0.01$.

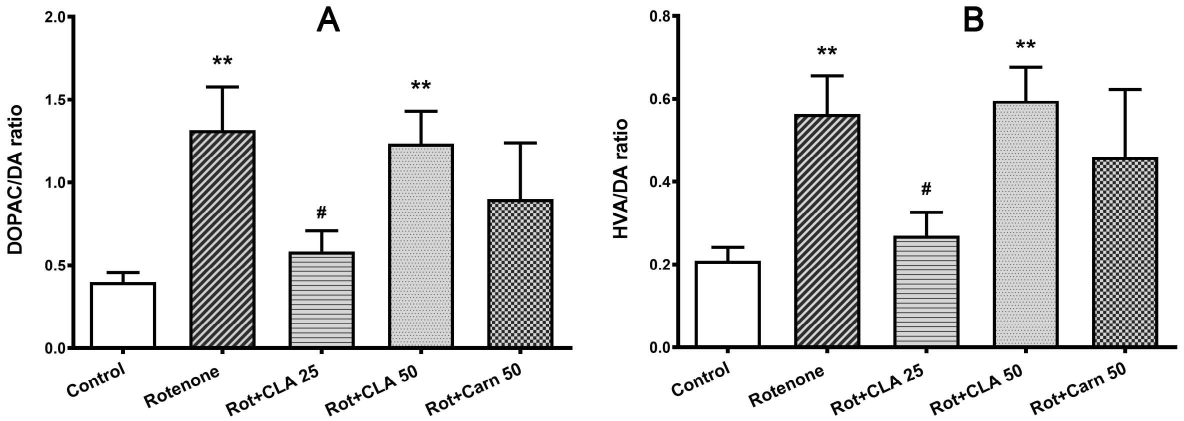

Figure S3. Effect of subcutaneous administration of rotenone, accompanied by the administration of carnosine (50 mg/kg) or carnosine-lipoic acid (CLA) complex (CLA) (25 and 50 mg/kg) for 18 days on the ratios metabolite/dopamine: A - DOPAC/DA, B – HVA/DA in the striatum of experimental animals. * – differences from intact animals with $p < 0.05$, ** - $p = 0.01$; # - differences from animals that received rotenone with $p < 0.05$.

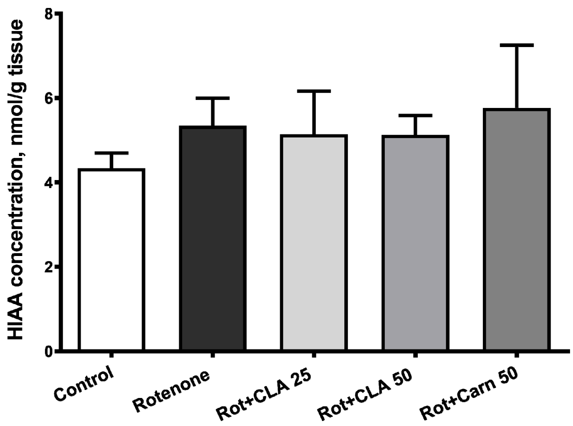

Figure S4. Effect of subcutaneous administration of rotenone, accompanied by the administration of carnosine (50 mg/kg) or carnosine-lipoic acid (CLA) complex (CLA) (25 and 50 mg/kg) for 18 days on the content of 5-HT metabolite 5-hydroxy indoleacetic acid (HIAA).
